# Genetic diversity and phylogenetic analysis of Crimean-Congo Hemorrhagic Fever viruses circulating in Pakistan during 2019

**DOI:** 10.1371/journal.pntd.0008238

**Published:** 2020-06-29

**Authors:** Massab Umair, Adnan Khurshid, Muhammad Masroor Alam, Ribqa Akhtar, Muhammad Salman, Aamer Ikram

**Affiliations:** Department of Virology, National Institute of Health, Islamabad, Pakistan; University of Texas Medical Branch, UNITED STATES

## Abstract

Being an endemic country for Crimean Congo hemorrhagic fever (CCHF), this study aimed to explore the genetic diversity of CCHF virus (CCHFV) detected in Pakistan during 2019. Serum samples from patients with clinical signs of hemorrhagic fever attending tertiary care hospitals in Pakistan were tested for CCHFV RNA using real-time PCR at Department of Virology, National Institute of Health. The partial S-gene fragments were directly sequenced to determine the prevailing CCHFV genotypes and their molecular epidemiology in Pakistan. During January-December 2019, 280 samples from suspected CCHF patients were tested and 28 (10%) were found positive on real-time PCR. Positive cases were detected from 14 districts and across all four provinces of Pakistan with majority reported during August-September. The mean age of CCHFV positive patients was 37.25 years (range 5–65 years) with a high frequency in males (92.8%; n = 26) and a case fatality rate of 40.7% was observed. Phylogenetic analysis showed that S- segment of 2019 PAK CCHFV strains (n = 13) belonged to Asia-1 genotype and clustered with regional strains from Iran, Oman, and Afghanistan. We conclude that Asia-1 genotype of CCHF virus remains endemic in Pakistan. Our findings emphasize to establish a laboratory based surveillance program to monitor the disease burden and identify outbreak hotspots for effective control.

## Author summary

Crimean-Congo hemorrhagic fever, a tick-borne viral disease is a zoonotic priority disease in Pakistan. During the current study, we found a mortality rate of 40.7% due CCHF which is high in comparison to studies from regional countries Iran, Afghanistan and Oman. The disease was commonly associated with people involved in animal husbandry and farming however other occupations were also affected. High proportion of cases was observed in the summer months, around the Eid-Al-Ahda festival. Genetic analysis revealed Pakistani CCHF viruses to be closely related with strains from bordering countries Iran and Afghanistan as well as with Oman indicating that animal trade and movement of people serve as a common source for the disease transmission.

## Introduction

Crimean-Congo hemorrhagic fever (CCHF) is a tick-borne zoonotic viral disease that was first recognized in 1944 in Crimea and later in 1969 in Congo thus given the name after its place of origin. The causative agent of the disease is Crimean-Congo hemorrhagic fever virus (CCHFV) which belongs to the *Nairovirus* genus in the *Bunyaviridae* family. The genome of CCHFV consists of single-stranded negative sense RNA that is divided into small (S), medium (M) and large (L) segments. The small and medium segments encode structural proteins nucleocapsid and glycoprotein respectively whereas the large segment encodes non-structural protein RNA dependent RNA polymerase [1, 2].

CCHFV is transmitted through Ixodid ticks (mainly of genus *Hyalomma*) which serve as reservoir and vector for the virus as well as through contact with blood or tissue from an infected human or animal. Although infected animals are asymptomatic, clinical features in humans include fever of abrupt onset, headache, myalgia, and thrombocytopenia however, complications can lead to hemorrhage, multiorgan failure and death [3, 4]. The geographic range of CCHFV is spread over 30 countries across Asia, Africa, Europe and the Middle East [5]. Based on phylogenetic analysis, CCHFV is classified into 7 genetic groups: two Asian, two European and three African [6] that correlate to geographic origin.

CCHF is among the list of zoonotic priority diseases for surveillance and response in Pakistan along with brucellosis, anthrax, salmonellosis, influenza, and rabies. Geographic location (bordering with CCHF endemic countries Afghanistan and Iran), climatic conditions that is favorable for vector amplification and unawareness of the disease among the rural population dependent on livestock and agriculture are the major contributors in the continued circulation of CCHFV in Pakistan. Although the virus was isolated from the ticks in the 1960s [7] the first human case of CCHF was recognized in 1976 during a laparotomy procedure on a patient admitted with abdominal pain, hematemesis, and melena in Rawalpindi, Pakistan. The outcome of the treatment was a nosocomial outbreak in the hospital staff resulting in three fatalities including a surgeon [8]. Since 1976, several sporadic outbreaks of CCHF have been described in Pakistani population and during 2010–2014 a total of 286 cases with a case fatality rate (CFR) of 20–29% was reported [9]. An alarming rise in CCHF cases was observed in 2016 with 86 positive cases and 41% CFR [10]. Surveillance data from Field Epidemiology and Disease Surveillance Division (FE&DSD), National Institute of Health (NIH), Pakistan showed 55 cases in 2017 and 63 in 2018. The situation has not changed in 2019 where 75 cases were confirmed till 8 December [11]. Notably, two provinces of Pakistan i.e. Balochistan that shares border with Afghanistan and Iran and Sindh particularly Karachi city are endemic areas for CCHFV in Pakistan although the virus is also reported from other provinces.

In Pakistan two main facilities provide diagnostic services for CCHF, one is National Institute of Health, Islamabad and the other is Aga Khan University Hospital, Karachi. NIH working under the Ministry of National Health Services Regulations and Coordination, Pakistan serves as the country’s central referral laboratory receiving thousands of samples for the diagnosis of different infectious diseases including CCHF. Although detection of CCHFV in suspected samples at these facilities is carried out through molecular assays using real-time polymerase chain reaction however, data on the genotypic diversity of circulating viruses is scarce from Pakistan [12–14]. Therefore, the objective of the current study was to investigate the molecular diversity of indigenous CCHFV strains and perform their phylogenetic analysis.

## Results

**Fig 1:**
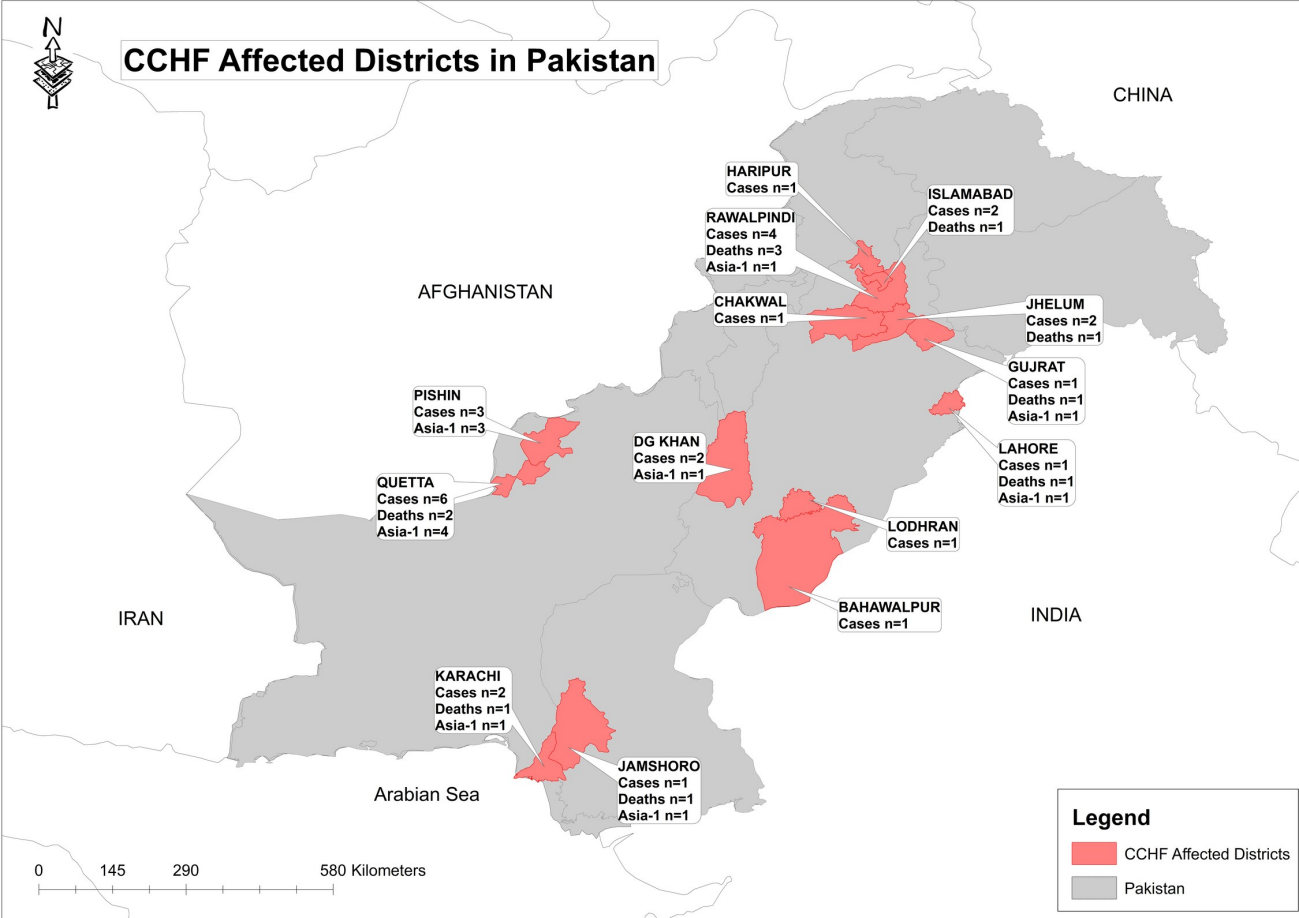
Geographical map of Pakistan showing CCHFV cases reported from the districts during 2019. The map was made using *Esri ArcMap 10*.*2* software.

**Fig 2:**
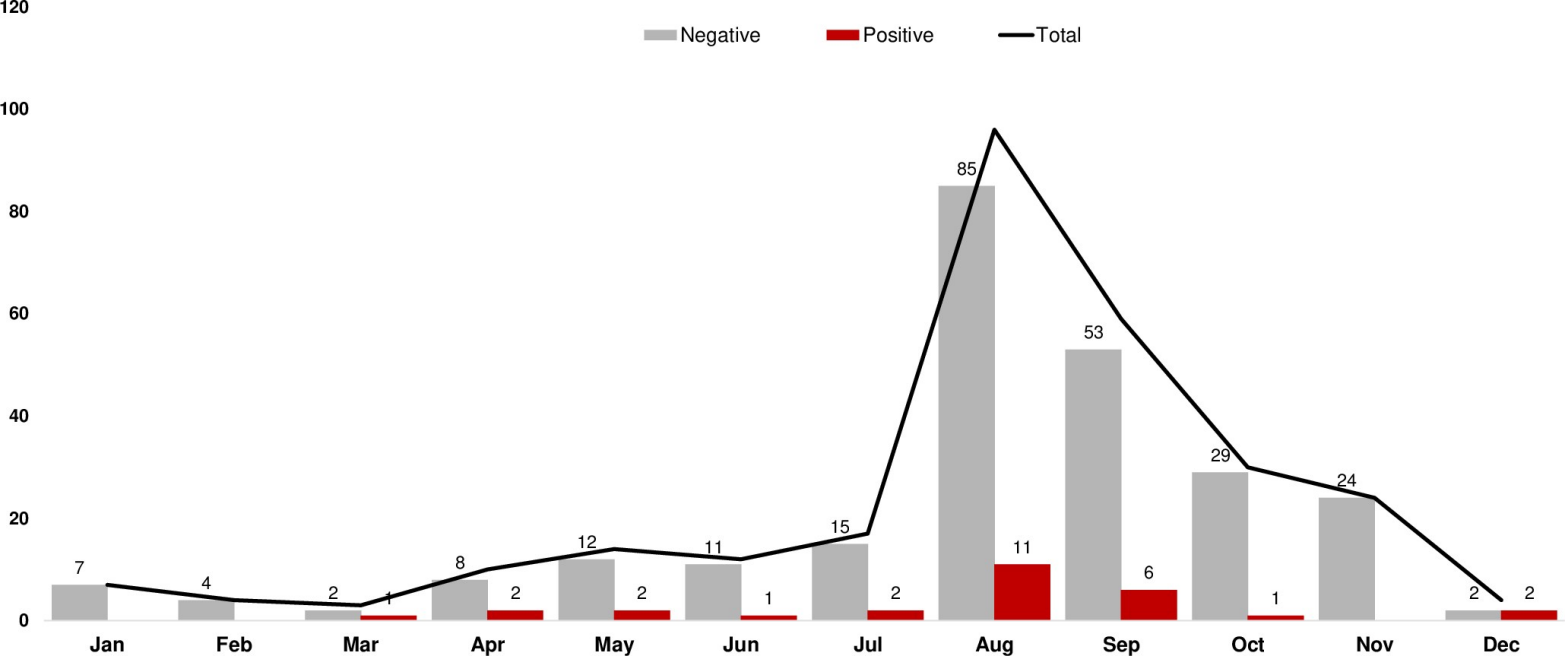
Month-wise distribution of CCHFV suspected and confirmed cases during 2019. Grey and red bars indicate number of negative and positive cases respectively. The total number of suspected cases are represented by grey line shown on secondary axis.

During 2019, a total of 280 blood samples from suspected patients of CCHF were referred to National Institute of Health, Islamabad for laboratory confirmation. On the basis of RT-PCR, 28 (10%) samples tested positive for CCHFV. Positive cases were detected from 14 districts and across all four provinces of Pakistan with most cases found in Quetta, Balochistan (Fig 1). Although CCHFV cases were reported from all the four seasons however, a peak was observed during summer i.e. August-September (Fig 2). Characteristics of the 28 patients diagnosed with CCHFV are presented in Table 1. The mean age of CCHFV positive patients was 37.25 years (range 5–65 years) with a high frequency in males (92.8%; n = 26). Clinical features in CCHFV patients included fever in 28 (100%), myalgia 12 (42.8%), vomiting 4 (14.2%), headache 3 (10.7%) hemorrhagic features 14 (50%) and other symptoms in 4 (14.2%). There were 11 deaths, resulting in a cumulative case fatality rate of 40.7% with 3 deaths reported from Rawalpindi, 2 from Quetta and 1 each from Islamabad, Gujrat, Jehlum, Lahore, Jamshoro and Karachi. The distribution of CCHFV cases by occupation showed that people involved in animal husbandry, farming and students were the most commonly affected (n = 3 each) followed by shepherd, milkmen, laborer and house wives (n = 2 each). One positive case was reported each from other occupations such as butcher, cattle handler, cobbler, driver, military person and retired government servant.

**Table 1:** Characteristics of laboratory confirmed (RT-PCR) cases of CCHFV during 2019.

Patient no.	Gender	Age (years)	Month admitted	Sign and symptoms	Occupation/ Profession	Residence (District)	Outcome
1	M	54	Mar	Fever and Nose bleeding	Animal husbandry	Karachi	Recovered
2	M	05	Apr	Fever and Nose & mouth bleeding	Not available	Jamshoro	Death
3	M	40	Apr	Fever and Nose bleeding	Animal husbandry	DG Khan	Recovered
4	M	41	May	Fever, Vomiting, Itching, Body rashes	Military	Lodhran	Recovered
5	M	20	May	Fever and Nose bleeding	Animal husbandry	DG Khan	Recovered
6	M	65	Jun	Fever, Hematemesis, Bleeding, Fritz	Farmer	Jehlum	Death
7	M	44	Jul	Fever and Nose & mouth bleeding	Milkman	Rawalpindi	Death
8	M	60	Jul	Fever, Vomiting and Mouth bleeding	Farmer	Gujrat	Death
9	F	30	Aug	Fever, Nausea, Vomiting and Headache	Housewife	Rawalpindi	Death
10	M	46	Aug	Fever, Abdominal pain and Myalgia	Butcher	Lahore	Death
11	M	19	Aug	Fever and Mouth bleeding	Student	Karachi	Death
12	M	28	Aug	Fever and Myalgia	Not available	Rawalpindi	Recovered
13	M	32	Aug	Fever, Myalgia and Headache	Farmer	Pishin	Recovered
14	M	65	Aug	Fever and Myalgia	Retired govt. servant	Quetta	Death
15	M	60	Aug	Fever and Myalgia	Not available	Quetta	Death
16	M	16	Aug	Fever, Myalgia and Headache	Cobbler	Pishin	Recovered
17	M	18	Aug	Fever and Myalgia	Student	Quetta	Recovered
18	M	49	Aug	Fever and Myalgia	Laborer	Pishin	Recovered
19	M	16	Aug	Fever and Myalgia	Student	Quetta	Recovered
20	M	60	Sep	Fever, Gum bleeding and Epistaxis	Shepherd	Chakwal	Recovered
21	M	20	Sep	Fever, Myalgia and Melena	Restaurant worker	Bahawalpur	Recovered
22	M	33	Sep	Fever, Abdominal pain and Hematemesis	Laborer	Islamabad	Death
23	M	65	Sep	Fever and Mouth bleeding	Driver	Haripur	Recovered
24	F	35	Sep	Fever and Myalgia	House wife	Quetta	Recovered
25	M	35	Sep	Fever and Myalgia	Not available	Quetta	Recovered
26	M	27	Oct	Fever, Vomiting and Hematuria	Milkman	Rawalpindi	Death
27	M	40	Dec	Fever, Melena and Hematemesis	Cattle handler	Islamabad	Recovered
28	M	20	Dec	Fever and Melena	Shepherd	Jhelum	Recovered

**Fig 3:**
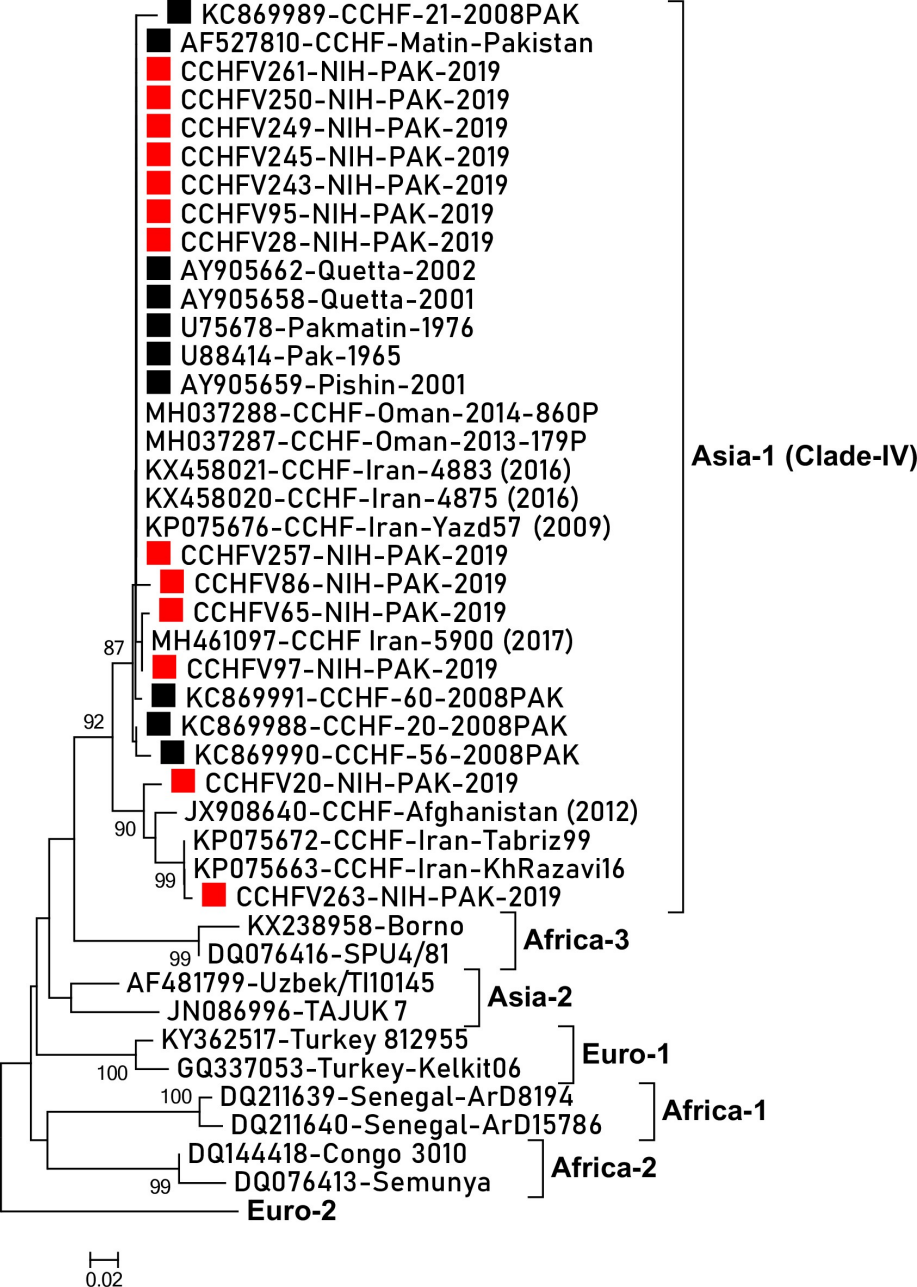
Phylogenetic analysis of CCHF viruses based on partial S-gene segment. The phylogenetic tree was reconstructed using neighbor joining method (bootstrap values 1000 replicates). Filled green squares (green) represent viruses from current study while filled circles (black) indicate viruses detected from Pakistan previously.

The partial S-gene segment of CCHF viruses was sequenced to determine their genetic diversity and phylogenetic relationship with strains circulating worldwide. All Pakistani CCHFV strains sequenced in the current study (n = 13) showed 91.7–100% nucleotide and 98.5–100% amino acid similarities. When compared with previous CCHFV strains reported from Pakistan, our viruses showed a 91.6–100% nucleotide and 95.5–100% amino acid resemblance. Pakistani viruses differed from previously reported indigenous strain KC869989 at amino acid positions 114 (Serine>Phenylalanine) & 115 (Lysine>Glutamic acid) and from KC869990 at 153 (Glutamic acid>Lysine). One amino acid change at amino acid position 155 (Valine>Isoleucine) was detected in strain CCHFV86-NIH-PAK-2019 compared to Pak-Matin reference virus (accession number: AF527810). Phylogenetic analysis based on partial S-gene sequence revealed clustering of Pakistani viruses into Asia-1 genotype (clade IV) along with strains reported from neighboring countries Iran, Afghanistan and Oman with 98.5–100% amino acid similarities (Fig 3).

## Discussion

The present report summarizes the epidemiological and genetic analysis of CCHF viruses detected in blood samples of clinically suspected and subsequently laboratory confirmed cases during January to December, 2019. The study subjects include patients from 14 districts across the four provinces of Pakistan that were admitted to different tertiary care hospitals.

Previously, a bi-annual surge of CCHF cases has been reported from Pakistan with majority distributed during March-May and August-October [14, 15]. In contrast, the present study showed a single peak (August-September) during 2019 however, this finding is in concurrence with a nationwide surveillance of CCHF carried out in 2016 [10]. The role of Eid-al-Adha (a religious festival celebrated annually by Muslim’s with slaughtering of animals at mass levels) in the spread of CCHF in Pakistani population is well discussed [10, 16, 17]. The festival was celebrated during 11–15 August 2019 in Pakistan, the month where we reported the highest number of laboratory confirmed cases of CCHF. However, Eid-al-Adha may not be the primary factor contributing to the high frequency of the disease rather we believe that climatic conditions of summer which favor tick proliferation to be the main reason which is supported by the high abundance of hyalomma ticks during the season [18]. Moreover, a comprehensive seven year data (2011–2017) on the prevalence of CCHF in Pakistan revealed that no direct association exists between time of Eid-al-Adha and occurrence of CCHF cases [17]. Awareness of CCHF disease pattern in Pakistan is imperative from the point of view of outbreaks as it will assist health authorities to target their limited resources for preventative measures during these months.

Although the first case of CCHF was reported in 1976 from Pakistan, lack of a surveillance system and limited diagnostic facilities across the country are main obstacles in determination of disease burden and fatality. A ten year study conducted during 1995–2005 in Balochistan disclosed a 15% mortality in patients due to CCHF [19]. Another study carried out in the same region during 1997–2002 involving 83 laboratory confirmed CCHF patients revealed a mortality of 9.6% [15]. During 2003–2006, 42 deaths (12.8%) were reported due to CCHF across Pakistan [9]. Similarly, a study involving the investigation of CCHF in patients belonging to 5 districts of Balochistan during 2011 showed a mortality rate of 20.4% [14]. CCHF cases reported in 2012 and 2013 from Pakistan showed case fatality rates of 29% and 20% respectively with Balochistan, Khyber Pakhtunkhwa and Punjab as the most affected provinces [20]. Notably, our study demonstrated a high mortality (40.7%) compared to previous reports however, this finding is in agreement with a prospective study carried out in 2016 that included CCHF patients admitted to hospitals located across all provinces of Pakistan and showed a mortality of 41% [10]. Representative case fatality rates from neighboring countries include 17.6% in Iran, 15% in Afghanistan and 36.4% in Oman [21–23]. The high fatality rates observed in our study and in the country generally could be due to late presentation at health facilities, non-standardized treatment protocols, scarcity of diagnostic laboratories and lack of awareness in general public about the disease, its prevention and control.

Occupational vulnerability to CCHF for people involved in farming, animal handling, veterinary, slaughtering of animals and healthcare has been documented [24]. Most patients infected with CCHF in our study belong to animal husbandry, farming, dairy along with students and housewives. Pakistani rural population is mainly dependent on animal husbandry and farming for living in which males are more commonly involved. Hence, gender susceptibility of the men for CCHF infection (92.5%) was observed in the present study. Possible risk factors in our study were occupational as most cases were involved in animal husbandry and farming and may have had contact with infected ticks and/or animals. However, factors which led to infection in patients with other occupations such as laborer, student and housewife etc. could not be ascertained. Previous investigation of CCHF patients admitted in a tertiary care hospital in Karachi showed most cases were students and butchers [25]. Data from a CCHF endemic and neighboring country Afghanistan revealed housewives, health staff, shepherds and butchers to be the high risk occupational groups [26]. Notably, in the present study no case of CCHF was observed in healthcare personnel suggesting good awareness about the disease and strict implementation of infection prevention and control in such settings. A major challenge to reduce CCHF infection in Pakistani population will be to create awareness of risk factors and educate people about preventive measures since the high risk population (farmers, nomads and animal handlers) lives in remote rural areas and are illiterate.

Limited genetic information of indigenous CCHF viruses is available from Pakistan and initial report of samples collected between 1998–2002 which were referred to international laboratory (National Institute for Communicable Diseases, South Africa) identified Asia-1 genotype having high similarities with CCHF viruses from Iran [27]. With the establishment of sequencing facility at national level, CCHF samples collected in 2008 and 2011 from Balochistan province demonstrated the presence of Asia-1 genotype in indigenous population [12, 14]. Molecular analysis of strain CCHF-43-2008PAK isolated from a patient admitted in Fatima Jinnah General and Chest hospital, Quetta in 2008 showed the cause of infection to be Asia-2 genotype [13]. Beyond the year 2011, no data is available on the genetic diversity of CCHF viruses from Pakistan despite the increasing number of cases [28]. The detection of Asia-1 genotype from distant geographic regions i.e. 8 districts and 3 provinces in our study further substantiates the endemicity of this genotype in the country. When subjected to phylogenetic analysis, Pakistani CCHF viruses were found to be highly related to strains from Iran, Afghanistan and Oman [23, 29]. The circulation of CCHF viruses in Pakistan that possess high S-segment similarities with Iranian strains suggests close transmission link between these bordering countries which is in consensus with previous reports [12, 30–32]. Pakistani CCHF viruses showed close homology with strains MH037287 & MH037288 isolated from Oman during 2013–2014 indicating frequent trade of livestock and cultural exchanges. In fact, 30% of Omanis are of Balochi origin from Pakistan's Balochistan province, being settled in Oman since many decades. Genetic similarities between Omani and Pakistani CCHF viruses has also been reported by Al-Abri, *et al*., [23]. Notably, Pakistani strains showed high nucleotide and amino acid similarities with strain JX908640 which was isolated from a 38 year old patient who visited his family in Samangan province of northwest Afghanistan in 2012 and then traveled back to United Kingdom where he later succumbed to the disease [33]. Tracing of CCHF viruses and any importations to their source requires robust sequencing and well established surveillance systems. Although our study was based on genetic analysis of partial S-gene sequence which has been previously used for accurate genetic classification of CCHF viruses into seven distinct genotypes [2, 12–14, 31, 34, 35], we recommend sequencing of complete S-segment in future to better understand the genetic diversity and possibility of recombination [36] in indigenous strains. To our knowledge, this is the first report of phylogenetic analysis of CCHF viruses circulating in distant geographic regions across Pakistan.

A major challenge in timely diagnosis of CCHF in Pakistan is the lack of diagnostic facilities at provincial levels let alone the districts. National Institute of Health, Pakistan situated in capital city serves as the National Public Health Institute and focal point for the implementation of International Health Regulations 2005 (IHR). The department of virology of this Institute provides support in diagnosis of CCHF from samples received from all parts of the country. Although laboratory testing of suspected CCHF cases is carried out on priority basis, transport of specimen to NIH from different regions is costly and time consuming which ultimately affects patient management. To strengthen national laboratory system that can address such challenges, NIH in collaboration with WHO and provincial health departments has planned to establish provincial public health laboratories in all provinces which will create and support regional capacities for diagnosis of infectious diseases including CCHF. Two of these laboratories have already been established in Khyber Pakhtunkhwa and Balochistan and public health laboratory of Balochistan has started testing and reporting of CCHF cases from the province thereby eliminating the need to send these samples outside the province. The fact that CCHF cases were reported from Punjab and Sindh during our study highlights the need for establishment of public health laboratories in these provinces on priority. In fact the findings of Joint External Evaluation (JEE) of IHR core capacities of Pakistan also highlighted the need to establish a strong, active surveillance and tiered public health laboratory system, covering human and zoonotic animal health with appropriate infrastructure, at national and provincial levels [37].

CCHF is a major public health issue in Pakistan. The virus which was confined to certain geographic regions of the country is now sporadically reported from all parts that are probably linked to the trade of livestock and frequent movement of nomadic population. Moreover, with the growing trend of animal trading and farming CCHF cases may increase in future thus warrants continuous monitoring and surveillance. To control CCHF in Pakistan, efforts are needed to create awareness about the disease among high risk groups, develop and strengthen diagnostic facilities, regulate the movement of animals especially during religious festival and strengthen coordination and collaboration between animal and human sectors. Accordingly, in future the role of Animal Quarantine Department (AQD) of the Ministry of National Food Security & Research and the National Veterinary Laboratory against the control of zoonotic diseases of trans-boundary in nature and of public health significance like CCHF will be critical.

## Methods

### Ethics statement

The study design was approved by the Internal Review Board of National Institute of Health, Pakistan.

### Sample collection

From January to December 2019, a total of 280 blood specimen collected from suspected CCHF patients admitted in different hospitals across the country were sent to the National Institute of Health, Islamabad for laboratory confirmation of CCHFV. The NIH, Pakistan defines a suspected CCHF case as a patient with sudden onset of fever over 38.5°C for more than 72 hours and less than 10 days, especially in a CCHF endemic area and those in contact with livestock such as shepherds, butchers, animal handlers and health care personals. A probable case is a suspected case with history of febrile illness for 10 days or less with epidemiological link and any two of the following: thrombocytopenia less than 50,000/mm, petechial or purpuric rash, epistaxis, hematemesis, hemoptysis, blood in urine and/or stools, ecchymosis and gum bleeding. While a confirmed case is a suspected/probable case confirmed through PCR and/ or serology [28]. Samples from different hospitals were sent to NIH in triple packaging under cold chain along with clinical and demographic data however, the individual identity was kept anonymous.

### Molecular testing and phylogenetic analysis

All the 280 samples were screened for the presence of CCHFV through real-time PCR. Viral RNA was extracted using the QIAamp viral RNA mini kit (Qiagen GmbH, Germany) according to the manufacturer’s instructions. Detection of CCHFV was carried out using the RealStar kit (Altona Diagnostics GmbH, Germany) following instructions in the manual. For the purpose of phylogenetic analysis, all positive samples on real-time PCR were further subjected to PCR amplification of partial S-segment as described previously [38]. Amplified PCR products were then directly sequenced on ABI 3100 genetic analyzer using Big Dye terminator cycle sequencing reaction kit v3.1 (Applied Biosystems, USA). CCHFV sequences were edited by sequencher software v.4.9. (Gene Codes Corp., USA) and analyzed with MEGA7 [39]. Sequences of CCHF viruses sequenced in the current study are submitted to the GenBank under the accession numbers MN839490—MN839497, MN839499, MN839500, and MN839502- MN839504.

## Supporting information

S1 Checklist STROBE checklist. (DOC)
